# Practical Notes on Diagnosis and Treatment

**Published:** 1906-10-06

**Authors:** 


### Practical Notes on Diagnosis and Treatment.

### Nematodes and Appendicitis.

Capt. H. W. Whate, M.B., reports a case in which all the classical symptoms of appendicitis disappeared after the patient had passed *per anum* twenty-four large round worms.

### Sciatica and Diabetes.

Sciatica, especially when bilateral and not dependent on disease within the pelvis, is suggestive of diabetes. It should also be remembered that the pains of tabes dorsalis are often described under such terms as sciatica or rheumatism.

### Unequal Pupils.

Inequality of the pupils is no doubt sometimes a congenital peculiarity; but, unless explained in this way, its existence, even as an isolated fact, justifies a suspicion of commencing cerebral nervous disease. That disease may be tabes dorsalis, or it may be general paralysis of the insane.—*Dr. Harry Campbell.*

### Antipyretic Measures in Phthisis Pulmonalis.

Before resorting to drugs, cold sponging and the application of cold compresses should be given a somewhat extended trial. The temperature should be taken every three hours, and when a rise to or above 102° F. occurs, the surface of the body should be sponged with ice-water and a cold compress applied over the thorax and abdomen. Niemeyer's pill of quinine, digitalis, and opium may at the same time be taken three times a day.—*Dr. H. Hyslop Thomson.*

### Syphilis and Heart Disease.

Gummata of the heart are rare, but a large proportion of the cases of fibroid disease of the heart depend on syphilis. The fibrosis is found principally in the inter-ventricular septum, and the condition carries a peculiar liability to sudden death. Aortic valve disease occurring apart from mitral disease may be attributed to atheroma or strain or syphilis. Hence, when this condition exists in women it is strong evidence of syphilis. Rheumatism may be suggested as an alternative diagnosis, but it is rare for the aortic valve alone to be affected by rheumatism.—*Dr. Hale White.*

### Catheter Lubricant.

The following formula is advised by Dr. Kraus: Tragacanth 25 grammes, glycerine 10 grammes, aqueous solution of carbolic acid 90 grammes. These, when triturated together in the cold, form a thick syrup. Besides its antiseptic virtues, this lubricant has a soothing effect on mucous membrances, and as it is soluble in water catheters smeared with it are readily cleansed. It is also a suitable lubricant to commit to patients themselves or to nurses when the catheter has to be used in the absence of the surgeon. It acts very well, too, as a lubricant for the finger in making rectal or vaginal examinations.

### Strabismus in Children.

It is important to remember that the sight of a squinting eye depreciates, and that, therefore, delay in treatment is dangerous. The first step is to estimate and correct the refraction, and there is no difficulty in teaching even the youngest child to wear spectacles. In addition, the defective eye must be educated. Hence the sound eye should be kept covered with a pad of wool arranged under the spectacle glass. The notion that treatment should be delayed until the child is a certain age is often responsible for complete loss of vision in the squinting eye. Operation is indicated only after failure of the above plan.—*Mr. Priestly Smith.*

### Counter Irritation in Ocular Disease.

In keratitis most gratifying results are often obtained by following the old-fashioned plan of blistering the eyelids with solid caustic; and in iritis, when the disease is tending to relapse time after time, the application of a blister often brings about such a change that recovery goes on afterwards without interruption. A still more pronounced result is obtained if the blistered surface be kept open by the application of an irritating ointment. When the inflammation is due to syphilis the presence of an open sore is most helpful, and therefore in such instances I have not the slightest hesitation in inserting a seton in the nape and keeping it there for several months.—*Dr. Maitland Ramsay.*

**Diet in Diabetes.**

Many deviations from the hard and fast lines of dietetic treatment are required in practice. Nevertheless, reduction in the glycosuria to its lowest possible limit must be the goal that is never lost sight of. Deviation from this rule can be allowed only when the dietetic measures taken to reach it are evidently producing worse evils, and even then we must hope to return at a later date to the more stringent measures. The practitioner who has not this end in view cannot be regarded as providing the proper treatment for cases of diabetes. Only in quite hopeless cases may such an aim be forgotten.—*Professor van Noorden.*

